# Lack of Association of Serum MMP-9 Concentration and rs17576 Single Nucleotide Variant *MMP-9* Gene With the Degree of Coronary Atherosclerosis and Other Risk Factors in Ukrainian Patients With Coronary Artery Disease

**DOI:** 10.1155/crp/6610742

**Published:** 2025-02-23

**Authors:** Oksana S. Pogorielova, Viktoriia V. Korniienko, Yaroslav D. Chumachenko, Olha A. Obukhova, Yelizaveta A. Stroy, Viktoriia Yu Harbuzova

**Affiliations:** Academic and Research Medical Institute, Sumy State University, Sumy, Ukraine

**Keywords:** coronary artery disease, glomerular filtration rate, matrix metalloproteinase-9, polymorphism

## Abstract

This study probes the relationship between serum matrix metalloproteinase-9 (MMP-9) levels, the genetic variant rs17576 in the MMP-9 gene, and the extent of coronary atherosclerosis among Ukrainian patients diagnosed with coronary artery disease (CAD). A cohort of 128 patients was assessed, comprising 25 with angiographically intact (normal) coronary arteries, 40 with acute coronary syndrome (ACS), and 63 with chronic coronary syndrome (CCS). Utilizing clinical, anthropometric, and biochemical analyses, alongside ELISA immunoassays, genotyping, electrocardiography, and coronary angiography, we conducted a comprehensive evaluation. Our findings indicate that MMP-9 levels peaked in ACS patients, particularly those with single and triple-vessel coronary lesions, while the lowest levels were observed in individuals with unaltered coronary arteries. Notably, the glomerular filtration rate (GFR) was highest in patients with angiographically normal coronary arteries, averaging 79.91 ± 27.8 mL/min. In the context of ACS, individuals carrying the GG allele exhibited the highest GFR, whereas AA allele carriers had the lowest. Conversely, in the CCS cohort, GG carriers demonstrated the lowest GFR and heterozygotes the highest, although these differences did not reach statistical significance. A significant disparity in serum MMP-9 levels was observed between ACS patients, CCS patients, and individuals with unimpaired coronary arteries. Moreover, a substantial correlation was established between the degree of coronary artery lesions and GFR in the CCS group, providing a predictive measure for GFR in patients with triple-vessel involvement. However, no significant association was detected between serum MMP-9 levels, the rs17576 genetic variant in the MMP-9 gene, and the number of affected vessels or GFR in both ACS and CCS patients.

## 1. Introduction

Coronary artery disease (CAD) is a leading cause of mortality worldwide, accounting for a considerable proportion of deaths in Europe [1]. The condition's multifactorial etiology encompasses a variety of genetic and environmental factors. Risk factors such as dyslipidemia, diabetes, hypertension, and obesity are well-established contributors to the initiation and progression of CAD, which is primarily underpinned by atherosclerosis [2]. Lifestyle factors, including sedentary behavior and poor diet, along with demographic shifts such as an aging population, have exacerbated the risk of atherosclerosis in Ukraine.

Inflammation's critical role in atherosclerosis and CAD has been affirmed by extensive research, emphasizing its contribution to the swift progression of arterial injury and arteriosclerosis [3, 4]. Matrix metalloproteinase-9 (MMP-9), a member of the matrix metalloproteinase enzyme family, has been implicated in atherosclerotic plaque destabilization. Notably, MMP-9's enzymatic activity in ECM metabolism, particularly within unstable atherosclerotic plaques, is significant [5–7]. Furthermore, MMP-9 expression levels correlate with plaque vulnerability and could serve as a predictive marker for plaque instability and the likelihood of adverse cardiovascular events [8, 9].

Our prior research indicated that while MMP-9 concentration may not predict acute myocardial infarction (MI) risk in the Ukrainian CAD population, carriers of the AG allele of the rs17576 single nucleotide variant (SNV) in the MMP-9 gene exhibited a reduced MI risk [10]. To further elucidate the impact of the MMP-9 (rs17576) SNV on the degree of coronary atherosclerosis, we examined various CAD-associated risk factors.

Accordingly, the aim of this study was to investigate the associations between serum MMP-9 levels, the MMP-9 (rs17576) SNV, the extent of coronary atherosclerosis, and other recognized risk factors in a cohort of Ukrainian CAD patients. Our findings are intended to enhance the understanding of CAD pathophysiology and inform potential strategies for therapeutic intervention.

## 2. Materials and Methods

### 2.1. Subjects

This study forms part of broader research evaluating risk factors associated with CAD in the Ukrainian population, concentrating on the interplay between serum MMP-9 levels, the rs17576 variant of the MMP-9 gene, CAD manifestations, and prognostic indicators. This phase of the research continues from work previously reported [10].

We enrolled 128 participants as outlined in our prior publication [11]. The study inclusion criteria were adults aged ≥ 18 years, diagnosis of CAD confirmed by coronary angiography (CAG) and for CCS—documented stable angina or MI in anamnesis. Exclusion criteria included presence of any acute infection or inflammatory disease, recent surgery or trauma, chronic inflammatory conditions or autoimmune diseases, pregnancy or lactation, and renal failure or undergoing dialysis. The participants were divided into the following: Group 1 with 25 individuals showing angiographically normal coronary arteries (CAs); Group 2 with 40 individuals presenting with acute coronary syndrome (ACS); and Group 3 with 63 patients exhibiting symptoms of chronic coronary syndrome (CCS). In this study, CCS includes patients with stable angina of functional classes 2-3 or with a history of MI. The main indication for CAG was presenting with symptoms indicative of CAD, such as chest pain (angina), shortness of breath, or other related symptoms, particularly if noninvasive testing suggests ischemia or patients with ACS.

The study took place at the Sumy Regional Cardiology Hospital in Sumy, Ukraine, following the Declaration of Helsinki's ethical standards. Approval was granted by the Ethics Committee of the Medical Institute at Sumy State University, with all participants providing written informed consent. Data collected included demographic and clinical details, such as age, gender, weight, height, smoking status, and arterial hypertension (AH) presence. Body mass index (BMI) was calculated for all participants. After undergoing electrocardiography and CAG, participants were assigned to groups based on CA status, with ACS patients receiving immediate troponin testing upon hospital admittance. Blood samples for measuring lipid profile, glucose, fibrinogen, erythrocyte count, hemoglobin levels, and glomerular filtration rate (GFR) (estimated via the QxMD Calculator) were taken before CAG under standardized conditions.

### 2.2. ELISA Study

We drew whole blood samples into tubes without anticoagulants and allowed coagulation at room temperature for 30 min, followed by centrifugation at 3000 rpm and 4°C for 15 min. Serum was decanted into clean polypropylene tubes and stored at −80°C for subsequent MMP-9 level determination through a commercial ELISA kit (Platinum ELISA, Affymetrix eBioscience, BMS2016/2 MMP-9, Bender MedSystems GmbH), following the manufacturer's guidelines.

### 2.3. Genotyping

DNA was extracted from whole venous blood using the GeneJET Genomic DNA Purification Kit (Thermo Fisher Scientific, Lithuania). Genotyping of the rs17567 SNP in the MMP-9 gene was performed with Real-time PCR on the 7500 Fast Real-time PCR System (Applied Biosystems, Foster City, USA), using TaqMan SNP Assays (TaqMan SNP Assay C_11655953_10).

### 2.4. Statistical Analysis

Statistical computations were performed using SPSS software (Version 22.0, Chicago, IL, USA). The Kolmogorov–Smirnov test assessed the distribution normality of continuous variables. Nonnormally distributed continuous data were compared using the Mann–Whitney test. Categorical variables, allele, and genotype frequencies were evaluated with the chi-squared (χ^2^) test. Compliance with Hardy–Weinberg equilibrium for allele distribution was checked for each group (https://wpcalc.com/en/equilibrium-hardy-weinberg/). We explored associations between the rs17567 SNP in the MMP-9 gene, serum MMP-9 concentrations, and CAD development via logistic regression, considering dominant, recessive, overdominant, and additive genetic models. A *p* value < 0.05 was deemed statistically significant.

### 2.5. Limitation of the Study

This study has several limitations that should be considered when interpreting the results. First, the relatively small sample size may limit the generalizability of our findings. Second, while we have focused on serum MMP-9 concentrations and the rs17576 SNP of the MMP-9 gene, other genetic variants and biomarkers potentially influencing CAD progression were not explored. Additionally, the cross-sectional design of the study precludes the establishment of causal relationships between MMP-9 levels, genetic polymorphisms, and the severity of coronary atherosclerosis.

## 3. Results

In an extensive analysis of the demographic and clinical characteristics of patients with CAD, 103 individuals and 25 control subjects were examined, with their respective data being meticulously detailed in our previous research [11]. Within this cohort, age distribution was homogenous across all groups, with a male predominance noted. Significant differences were discerned in key clinical indicators among the groups, with a marked disparity in glucose levels, serum MMP-9 concentrations, and fibrinogen levels, all of which bear *p* values of 0.05 or less, indicating statistical significance. Notably, serum MMP-9 levels peaked in patients with ACS, registering at 660.65 ng/mL for those with one-vessel and 513.5 ng/mL for three-vessel CA lesions, indicative of a heightened inflammatory response. In stark contrast, patients with no CA lesions—a condition termed intact CA—showed the lowest serum MMP-9 levels. GFR, a critical measure of renal function, also varied, with the highest mean value of 79.91 ± 27.8 mL/min observed in patients with intact CA. These demographic and clinical profiles, in conjunction with the serum MMP-9 concentration variances, are collated and presented in Table 1, further reinforcing the lack of a statistically significant relationship between serum MMP-9 levels and the extent of coronary vessel affliction.

In an in-depth analysis of genetic predisposition among ACS patients, it emerged that half of the AA genotype carriers exhibited two-vessel CA lesions, and a striking 80% of individuals with the GG genotype were found in the same category. Notably, heterozygotes were predominantly affected by one-vessel lesions, accounting for 60% within this subset. This pattern was paralleled in the CCS cohort, where 60% of AG genotype carriers were identified with two-vessel lesions. However, the distribution among AA carriers lacked a definitive trend, and a significant proportion (23.5%) of GG carriers were discovered to have three-vessel involvements. Contrasting with the ACS group, heterozygotes with CCS were primarily impacted by one-vessel lesions, comprising 64.7% of their grouping. Despite these detailed observations, statistical analysis using Pearson's χ^2^ test revealed no significant disparities in the frequency distribution of genotypes across the groups studied, affirming the complexity of genetic influence on CAD progression, as substantiated by the results in Table 2.

The logistic regression analysis presented in our study did not reveal a significant connection between the number of CA lesions and the MMP-9 gene SNV in patients with ACS and CCS as outlined in Table 3. This finding invites a broader contemplation of the role of genetic factors in CAD progression. Echoing the insights of Duran et al. [12], who identified a decreased estimated glomerular filtration rate (eGFR) as an independent predictor of the severity of coronary lesions, our GFR data suggest a similar trend. Duran's research, which revealed the eGFR as a standalone risk factor influencing SYNTAX and Gensini scores along with age and male gender, aligns with our observations that the GFR was lowest in patients with extensive CA damage, as demonstrated in Table 4.

Furthermore, Kang, Jeong, and Kim's work [13] accentuates the significance of renal function in acute scenarios, highlighting renal dysfunction as an independent determinant of both mortality and ACS-related complications. This underscores the critical interplay between renal impairment and cardiovascular outcomes. Our investigation, which systematically measured GFR in ACS patients, revealed a consistent decline in renal function across the spectrum of CA lesion severity, signifying the potential of GFR as a marker for CAD progression. The measured GFR rates, indicative of stage II chronic kidney disease (CKD) according to KDIGO guidelines, reinforced the lack of direct association between vascular lesion extent and renal function within the acute context of ACS. Meanwhile, in the CCS cohort, the Kruskal–Wallis test showed significant differences in GFR, which varied directly with the number of affected vessels, suggesting a nuanced relationship between CA lesion extent and renal health over the longer term. This nuanced relationship is further corroborated by our statistical analysis which indicates a predictive capacity for GFR levels in patients with multivessel CA lesions (Table 4).

Investigations into the GFR and its relationship with vascular damage in patients with ACS revealed no significant correlations, thereby not supporting GFR as a marker for vascular damage in this acute setting. However, a different pattern emerges in CCS patients, where GFR values were statistically distinct across different degrees of CA lesions, as shown in Table 5. A linear regression formula (SEPI = 82.335 + (−16.188) = 66.147 mL/min) has been derived, enabling the prediction of GFR levels specifically for patients with extensive three-vessel CA lesions. This model underscores the potential utility of GFR as a prognostic tool in the context of CCSs.

In patients with ACS, those carrying the GG genotype exhibited the highest GFR with a mean value of 90.6 mL/min, whereas AA genotype carriers showed the lowest mean GFR of 68.75 mL/min. Conversely, in patients with CCS, individuals with the GG genotype presented with the lowest mean GFR of 70.65 mL/min, while the highest GFR was observed in heterozygotes. Nonetheless, these observations did not yield statistically significant differences in GFR with respect to the rs17576 SNV of the MMP-9 gene in cohorts with ACS and CCS, as determined by the Kruskal–Wallis test (Table 6).

The association between GFR and the MMP-9 gene SNV rs17576 was investigated utilizing a simple linear regression analysis. The analysis revealed no statistically significant correlation between the rs17576 SNV of the MMP-9 gene and GFR in patient groups with ACS and CCS, as indicated in Table 7.

## 4. Discussion

MMP-9, a member of the endoprotease family, is implicated in the remodeling of the vascular wall and in the formation of atherosclerotic plaques. Elevated levels of MMP-9 have been documented in the vasculature during ACS, as well as in diverse pathologies such as emphysema, arthritis, and certain progressive tumors [14]. Our research indicates that MMP-9 concentrations significantly vary across patient groups, with the highest levels observed in those with ACS and the lowest in those with intact CA. These findings are in line with previous studies, thereby supporting the role of MMP-9 as a marker for the progression and destabilization of atherosclerotic plaques [5, 14, 15]. The significant elevation of serum MMP-9 levels observed in patients with ACS compared to those with CCS and individuals with intact CAs was confirmed through statistical analysis. This finding reflects a heightened inflammatory response associated with the acute phase of CAD, consistent with previous studies. Our research highlights that MMP-9 levels are notably elevated in the ACS cohort, reinforcing its role as a potential biomarker for acute inflammatory processes in coronary syndromes.

Atherosclerosis is recognized as a systemic illness impacting arteries throughout the body. Comparative analysis of GFR in patients with intact CA and various forms of CAD revealed a significant correlation between GFR and the extent of vascular involvement in patients with CCS. However, this association was not evident in the ACS cohort, potentially due to the nature of acute CA occlusion not mirroring the extent of occlusion found in renal arteries. In CCS, prolonged atherosclerotic development in both cardiac and renal vessels can lead to compromised renal perfusion and reduced GFR, indicating renal dysfunction, a condition described in the literature as chronic cardiorenal syndrome. This association between the severity of CA lesions and renal function deterioration has been corroborated by various studies [16–18]. Recognizing the systemic impact of atherosclerosis, in 2003, authorities such as the American College of Cardiology/American Heart Association and the National Kidney Foundation classified patients with CKD as high cardiovascular risk [19].

GFR's decline can be attributed to a reduction in the number of functional nephrons due to renal parenchymal diseases, age-related changes, and diminished renal perfusion resulting from atherosclerotic renal artery lesions. Consequently, GFR typically diminishes by about 8 mL/min per decade after the age of 30. Angiographic studies have linked decreased renal function to the severity of CAD, independently of other risk factors [20]. The COURAGE cohort study further substantiated that three-vessel CA lesions are more prevalent in patients with CKD than in those without [21].

The human MMP-9 gene, positioned on chromosome 20q12.2–13.1, comprises 13 exons and 12 introns. Investigations into several common SNVs of the MMP-9 gene in relation to CAD have been conducted. Among them, the R279Q (rs17576) SNV, situated in exon 6 and involving an A to G substitution, results in an amino acid switch from arginine (R) to glutamine (Q) and a subsequent reduction in the enzyme's catalytic activity [22]. An aggregate of 7 case-control studies involving 5525 cases and 2497 controls has examined the MMP-9 (R279Q) SNV in the context of CAD risk [23, 24]. Despite variable and occasionally conflicting findings across different populations, one study associated the 279Q allele with increased MMP-9 levels and a higher incidence of cardiovascular mortality and nonfatal MI [6]. Yet, other studies have not found a correlation between the R279Q variant and CAD risk or stable angina [22–26]. The frequency of the rs17576 SNV in the European population is reported to be 0.355310 [27]. Our prior research did not establish a statistically significant link between the rs17567 polymorphism of the MMP-9 gene and the development of CCS or ACS (Pa > 0.05). However, logistic regression analysis suggested that AG carriers have a reduced risk of MI development in both crude (Pc = 0.033; ORc = 0.359; 95% CI = 0.14–0.922) and adjusted models (Pa = 0.023; ORa = 0.299; 95% CI = 0.106–0.848) following an overdominant pattern of inheritance [28]. Consistent with these findings, the current study also did not demonstrate a statistically significant association between the rs17576 SNV of the MMP-9 gene and the extent of CA lesions or GFR.

## 5. Conclusions

In summary, our study has established a significant elevation of serum MMP-9 levels in patients with ACS as compared to those with CCS and individuals with intact CA. While our data affirm a statistically significant association between the severity of CA lesions and GFR in patients with CCS, this relationship was not mirrored in the context of serum MMP-9 concentrations. Importantly, our findings demonstrate the ability to anticipate GFR levels in patients with tri-vessel coronary affliction, contributing to a more comprehensive prognostic framework. Despite the absence of a direct correlation between serum MMP-9 concentrations and the extent of vessel involvement and GFR, our research has unveiled an association between the rs17576 SNV of the MMP-9 gene and the degree of angiographically confirmed CA lesions as well as GFR in patient cohorts with both ACS and CCS. These insights underscore the nuanced role of the MMP-9 gene variation in the pathophysiology of CAD and its systemic manifestations, aligning with our investigative aim to elucidate the interplay between genetic factors and clinical outcomes in cardiovascular pathology. Therefore, the conclusions drawn from our investigation not only reinforce the potential of MMP-9 as a biomarker in coronary syndromes but also spotlight the significance of its genetic variations in influencing disease progression and patient prognosis. This dual focus consolidates our understanding of the genetic underpinnings that may guide future therapeutic strategies and personalized medicine approaches.

## Figures and Tables

**Table 1:** MMP-9 concentration in patients with different degrees of CA lesions.

Group	One-vessel lesion	Two-vessel lesions	Three-vessel lesions	*p*
ACS	660.65 (299.2–885.88)	205.3 (128.5–489.6)	513.5 (396.9–786.93)	0.111
	(*n* = 6)	(*n* = 15)	(*n* = 8)	

CCS	294.35 (145.13–566.53)	320.05 (106.41–545.63)	404.45 (203.28–743.88)	0.639
	(*n* = 16)	(*n* = 12)	(*n* = 12)	

**Table 2:** Distribution of genotype frequencies by rs17576 MMP-9 SNV in patients with different degrees of CA damage.

Genotype	ACS				CCS			
	The number of affected vessels			*χ* ^2^ (P^1^)	The number of affected vessels			*χ* ^2^ (P^2^)
	І	ІІ	ІІІ		І	ІІ	ІІІ	
АА	4 (22.2)	9 (50)	5 (27.8)	6.191 (0.185)	9 (45)	5 (29.4)	7 (41.2)	5.528 (0.237)
AG	6 (50)	3 (25)	3 (25)		10 (50)	11 (64.7)	6 (35.3)	
GG	0 (0)	4 (80)	1 (20)		1 (5)	1 (5.9)	4 (23.5)	

*Note:* The absolute number of patients (and the percentage in parentheses) is displayed in the table. (P^1^) is for patients with ACS and (P^2^) for patients with XIX. The Numbers I, II, and II demonstrated one-, two-, or three-vessel CA lesions.

**Table 3:** Analysis of the association between MMP-9 SNV and the number of affected CA in patients with ACS and CCS.

Model	ACS				CCS			
	Р_с_	OR_c_ (95% CI)	P_a_	OR_a_ (95% CI)	Р_с_	OR_c_ (95% CI)	P_a_	OR_a_ (95% CI)
Dominant	0.395	0.524 (0.118–2.327)	0.357	0.373 (0.046–3.044)	0.481	1.5 (0.486–4.631)	0.689	1.292 (0.37–4.511)
Recessive	—	—	—	—	0.296	3.276 (0.355–30.271)	0.247	5.134 (0.322–81.742)
Superdominant	0.051	0.211 (0.044–1.005)	0.137	0.18 (0.019–1.721)	—	—	—	—
Additive^а^	0.122	0.286 (0.059–1.395)	0.189	0.219 (0.023–2.111)	0.683	1.275 (0.398–4.087)	0.923	1.066 (0.293–3.882)
	—	—	—	—	0.263	3.75 (0.371–37.948)	0.293	5.653 (0.224–142.932)

*Note:* Index “c” indicates the results of logistic regression without amendments; index “a” indicates the results of logistic regression after adjusting for age, sex, body mass index, the presence of hypertension, and smoking habits.

^a^The upper row shows the comparison results for genotypes AG and AA, and the lower one shows those for GG and AA.

**Table 4:** The quantitative value of GFR depending on the degree of CA damage.

Index	The degree of vascular damage in patients with ACS			Р^1^	The degree of vascular damage in patients with CCS			p^2^
	І (*n* = 9)	ІІ (*n* = 18)	ІІІ (*n* = 9)		І (*n* = 20)	II (*n* = 17)	III (*n* = 17)	
GFR (ЕРІ), mL/min	77.6 (52.55–93.9)	71.85 (48–90.78)	74.3 (49.05–84.3)	0.872	81.85 (69.38–98.25)	74.9 (58.5–86.15)	65.6 (51.1–76.15)	0.037

*Note:* GFR values in the form of median and interquartile range are given for each degree of vascular damage. I—one-vessel CA lesion, II—two-vessel CA lesion, and III—three-vessel CA lesion.

Abbreviation: GFR, glomerular filtration rate.

**Table 5:** Analysis of the association between GFR and the degree of damage of the CA.

Predictors in patients with ACS	Р^1^	Predictors in patients with CCS	Р^2^
II	0.691	II	0.134
III	0.612	III	0.008
Constant	< 0.001		< 0.001

*Note:* I—one-vessel CA lesion, II—two-vessel CA lesions, and III—three-vessels CA lesions. Р^1^—for patients with ACS; Р^2^—for patients with CCS.

**Table 6:** The quantitative value of GFR depending on the genotype by rs17576 SNV MMP-9 gene.

Patients with ACS			Р^1^	Patients with CCS			Р^2^
AA	AG	GG		AA	AG	GG	
68.75 (48.5–86.75)	77.6 (64.8–91.8)	90.6 (67.05–98.35)	0.212	74.25 (67.25–88.75)	76.7 (57.08–91.15)	70.65 (36.53–97.53)	0.866
(*n* = 16)	(*n* = 11)	(*n* = 5)		(*n* = 18)	(*n* = 22)	(*n* = 6)	

*Note:* Р^1^—for patients with ACS; Р^2^—for patients with CCS.

**Table 7:** Analysis of the association between rs17576 SNV *MMP-9* gene and GFR.

Predictors in patients with ACS	Р^1^	Predictors in patients with CCS	Р^2^
AG vs. AA	0.205	AG vs. AA	0.998
GG vs. AA	0.08	GG vs. AA	0.479
Constant	< 0.001		< 0.001

*Note:* Р^1^—for patients with ACS; Р^2^—for patients with CCS.

## Data Availability

The underlying data should be requested from the corresponding author.
